# The Arbuscular Mycorrhizal Fungus *Funneliformis mosseae* Alters Bacterial Communities in Subtropical Forest Soils during Litter Decomposition

**DOI:** 10.3389/fmicb.2017.01120

**Published:** 2017-06-20

**Authors:** Heng Gui, Witoon Purahong, Kevin D. Hyde, Jianchu Xu, Peter E. Mortimer

**Affiliations:** ^1^Key laboratory for Plant Diversity and Biogeography of East Asia, Kunming Institute of Botany, Chinese Academy of SciencesKunming, China; ^2^World Agroforestry Centre, East and Central AsiaKunming, China; ^3^Centre of Excellence in Fungal Research, Mae Fah Luang UniversityChiang Rai, Thailand; ^4^School of Science, Mae Fah Luang UniversityChiang Rai, Thailand; ^5^Department of Soil Ecology, UFZ-Helmholtz Centre for Environmental ResearchHalle, Germany

**Keywords:** arbuscular mycorrhizal fungi, soil bacteria, Illumina sequencing, litter decomposition, soil microbial community

## Abstract

Bacterial communities and arbuscular mycorrhizal fungi (AMF) co-occur in the soil, however, the interaction between these two groups during litter decomposition remains largely unexplored. In order to investigate the effect of AMF on soil bacterial communities, we designed dual compartment microcosms, where AMF (*Funneliformis mosseae*) was allowed access (AM) to, or excluded (NM) from, a compartment containing forest soil and litterbags. Soil samples from this compartment were analyzed at 0, 90, 120, 150, and 180 days. For each sample, Illumina sequencing was used to assess any changes in the soil bacterial communities. We found that most of the obtained operational taxonomic units (OTUs) from both treatments belonged to the phylum of Proteobacteria, Acidobacteria, and Actinobacteria. The community composition of bacteria at phylum and class levels was slightly influenced by both time and AMF. In addition, time and AMF significantly affected bacterial genera (e.g., *Candidatus Solibacter, Dyella, Phenylobacterium*) involved in litter decomposition. Opposite to the bacterial community composition, we found that overall soil bacterial OTU richness and diversity are relatively stable and were not significantly influenced by either time or AMF inoculation. OTU richness at phylum and class levels also showed consistent results with overall bacterial OTU richness. Our study provides new insight into the influence of AMF on soil bacterial communities at the genus level.

## Introduction

In terrestrial ecosystems, litter decomposition plays a crucial role in carbon and nutrient cycling, which consists of complex physical, chemical and biological processes (Berg, 2000). These processes are driven primarily by microorganisms in the soil using various enzymes to degrade different components of the litter (e.g., lignin, cellulose, and hemicellulose) (Lynd et al., 2002). Among these microorganisms, although soil saprophytic fungi are usually considered the major decomposers (Hanackova et al., 2015), soil bacteria also play a major role (Greaves, 1971; Purahong et al., 2015). For example, DeAngelis et al. (2013) found that many fast growing bacteria, including Alpha-, Beta-, and Gamma-proteobacteria, Clostridia and Bacteroidetes were the initial decomposers in tropical forest soils. In addition, Actinobacteria are able to degrade cellulose (Schellenberger et al., 2010; Stursova et al., 2012), and many other bacterial groups have also been found to be engaged in lignin decomposition (Masai et al., 2007; Bugg et al., 2011). A recent study provides evidence that complex litter decay processes are the result of a dynamic cross-kingdom, functional succession between fungi and bacteria (Purahong et al., 2016).

Previous studies on litter decomposition have focused on the role of soil fungal communities, especially saprophytic fungi (Kuramae et al., 2013; Persoh et al., 2013; Treseder et al., 2014; Hanackova et al., 2015). However, despite the fact that bacterial communities and non-saprophytic fungi co-exist in the soil, the interactions between these two groups and their effects on litter decomposition has been largely ignored. Arbuscular mycorrhizal fungi (AMF), from the phylum of Glomeromycota, is the most common mycorrhizal association between soil fungi and terrestrial plants, with about 70% of plants forming mycorrhizal symbioses with AMF (Smith et al., 1997). The essential function of this symbiosis is acquiring nutrients (e.g., N, P) through its external mycelium in exchange for carbohydrate from host plants’ photosynthate (Smith and Smith, 2011). Furthermore, AMF are known to have no saprophytic capacity, but they can affect litter decomposition, both positively (Hodge, 2001; Cheng et al., 2012; Herman et al., 2012; Koller et al., 2013), and negatively (Leifheit et al., 2015). Although the mechanism underlying this effect is unclear, AMF might impact litter decomposition through effects on other saprophytic microorganisms in the soil (Nuccio et al., 2013) or on soil structure (Rillig and Mummey, 2006). Soil bacteria can obtain C from the exudates released by mycorrhizal hyphae or by using the hyphae themselves as substrate (Qin et al., 2014). The plant–AMF–soil bacteria interaction likely occurs in the mycorrhizosphere: as defined by Oswald and Ferchau (1968), this is the surface area of the mycelium and consists of two components, the rhizosphere and hyphosphere, which provide niches for the growth of bacteria (Toljander et al., 2006). Additionally, AM fungi are able to enhance the activity of bacteria associated with soil N availability (for plants, fungi, and bacteria), either using raw organic material as a substrate or by influencing the plant N cycle (Saia et al., 2014). In a microcosm experiment, Hodge et al. (2001) reported that the presence of AMF increased the decomposition of complex organic matter and that AMF were able to influence litter decomposition by altering the activity of hyphosphere bacteria. Furthermore, Herman et al. (2012) also reported that AM fungal inoculation altered the C flow as a result of altering the soil microbial community.

However, using the same experimental design as Herman et al. (2012), Nuccio et al. (2013) found that the presence of AMF resulted in a change of about 10% of the relative abundance of the bacterial community. In addition, Welc et al. (2010) reported that AMF mycelium could suppress the soil bacterial community. Furthermore, many specific bacterial taxa are highly associated with AMF as they colonize and live on the surface of the hyphae (Cruz and Ishii, 2011; Iffis et al., 2014). For example, certain *Proteobacteria* and *Firmicutes* taxa have been documented as co-existing with AMF (Bonfante and Anca, 2009; Scheublin et al., 2010; Lecomte et al., 2011).

In this study, a microcosm with two units separated by an air-gap was used to study the interaction between the hyphae of *Funneliformis mosseae* and soil bacterial communities during litter decomposition. We applied high-throughput sequencing techniques to characterize the soil bacterial community during litter decomposition. Specifically, we aimed to investigate how *F. mosseae* temporally altered soil bacterial diversity (i) and community structure (ii) during litter decomposition. Based on past studies investigating the interaction between AMF and soil bacteria (Toljander et al., 2006; Herman et al., 2012; Saia et al., 2014), we hypothesize that *F. mosseae* will significantly alter the bacterial community by stimulating key bacterial groups associated with litter decomposition.

## Materials and Methods

### Materials Used in the Experiment

#### (1) AMF Inoculum

Arbuscular mycorrhizal fungi inoculum (*F. mosseae*) was provided by The Institute of Plant Nutrition and Resources, Beijing Academy of Agriculture and Forestry Sciences (Beijing, China). The inoculum contained approximately 1000 spores per 20 g application and the sterile *F. mosseae* spores were contained (pre-mixed) in a rock flour material (20 g).

#### (2) Soil

The soil (pH = 4.1) used in the microcosms was collected from a subtropical forest located in southwestern China (N 21° 31′ 42.13″, E 100° 29′ 41.87″). The top 5 cm of soil was collected after first removing the litter layer. The soil was sieved using a 2 mm mesh in order to remove any stones or root material. Soil properties have been published in Gui et al. (2017).

#### (3) Litterbags

Litterbags were made of 200 μm nylon mesh. The litter comprised dried leaves of *Calophyllum polyanthum* Wall. ex Choisy, an indigenous tree to Yunnan Province, and one of the dominant species from the forest used to collect the soil. The leaves were collected from nursery grown *C. polyanthum* saplings, and were oven dried at 65°C to a constant weight. Then the dried leaves (Total *N* = 1.44%) were cut into small pieces (*ca.* 5 mm^∗^5 mm), 2 g of which were put into the litterbags. Note that the purpose of the litterbags was to allow for the monitoring of the impact of AMF on litter decomposition, the results of which have been published separately (Gui et al., 2017).

#### (4) The Host Plant

*Trifolium repens* L. cv. Milkanova was selected as the host plant. The seeds of *T. repens* were sterilized before use.

### Experimental Setup and Validation

Our experiments were conducted in an acrylic microcosm unit, the design of which has been described in Gui et al. (2017). Briefly, the microcosm unit consisted of two compartments. The first compartment (Host), which was designed to pot the host plant, was filled with sterilized vermiculite and fine gravel (*ca.* 0.3 cm diameter), which was evenly mixed in a 1:1 ratio. In order to test how *F. mosseae* interacts with soils from a tropical forest, the soil was placed in the second compartment (Litter). A litterbag (5 cm^∗^5 cm) was buried in the soil at a depth of 5 cm so that the soil microbial community could fully interact with the surface of the litter. Even though this burying set-up could not exactly simulate what happens in the field, it did allow for the litter to be exposed to the microbial communities in the soil. The two compartments of the microcosm were separated by a plate, which was drilled with evenly spaced holes (4 mm in diameter) and covered by 20 μm nylon mesh on both sides, the mesh allows the hyphae of *F. mosseae* to pass through, but not plant roots (Supplementary Figure S1).

The microcosms were divided into two treatments, based on the host plant being inoculated with *F. mosseae* (AM) or not (NM). For NM control, the same amount of sterilized rock flour (20 g) was added into the host compartment. Four replicates were set for each treatment. Additionally, four time-phase samplings were taken at monthly intervals. Thus, in total, 32 microcosms were set up for the experiment. All the microcosms were randomly placed in a greenhouse with daily temperature ranging from 20 to 25°C. Plants received natural light only and no rainwater.

### Plant Growth and Percentage AMF Colonization

The 0.2 g of *T. repens* seeds were planted in the Host compartment of the microcosm (Supplementary Figure S1). The Host compartment received 10 ml of distilled water twice a week and 10 ml of modified Long Ashton nutrient solution once a week (Hewitt and Bureaux, 1966). The Litter compartment received 10 ml of distilled water once a week in order to maintain the moisture levels. After 2 weeks the N and P concentrations in the Long Ashton solution was diluted to 1/10 of the original concentration (34 mg⋅L^-1^ NaNO_3_ + 21.4 mg⋅L^-1^ NH_4_Cl, 29.2 mg⋅L^-1^ NaH_2_PO_4_⋅2H_2_O + 4.7 mg⋅L^-1^ Na_2_HPO_4_⋅12H_2_O) according to Leigh et al. (2009) and adjusted to pH 7.0 with NaOH.

The percentage of *T. repens* root length colonized by AMF was determined using fresh root samples. Root pieces (2 cm in length) were washed in distilled water and then rinsed with 10% KOH, which was stained with pen ink according to the methods of Vierheilig et al. (1998). The percentage colonization was calculated by a modified line intersection method (McGonigle et al., 1990).

### Soil Sampling and Nutrient Analyses

The original soil was collected from the forest as four subsamples which were bulked into one composite sample and then preserved at -20°C for the later analysis and this original soil was marked as “O.” The first time of sampling from the microcosm was conducted 90 days (T_90_) after planting, subsequent sampling times were at 120 days (T_120_), 150 days (T_150_), and 180 days (T_180_) after planting. Five grams of the soil around the litterbag was collected and preserved at -20°C for further DNA analysis.

Total soil organic carbon was determined by Dumas combustion (White et al., 1997), and total N using a semi-micro Kjeldahl apparatus (Yuen and Pollard, 1953). Total phosphorus (P) and potassium (K) were measured spectrophotometrically after digesting a mixture of concentrated H_2_SO_4_ and H_2_O_2_ (Murphy and Riley, 1986). Hydrolysable N was analyzed through a reaction with iron (II) sulfate and sodium hydroxide in a diffusion procedure (Mulvaney and Khan, 2001). Available P and K were determined using ammonium fluoride and ammonium acetate (Hedley et al., 1982). All the chemical analyses were conducted in Yunnan Agriculture Academy, Yunnan Province, China.

### DNA Extraction, Illumina Sequencing Analysis, and Data Processing

Total soil genomic DNA was extracted from 2 g of fresh soil using OMEGA Soil DNA kit, following the manufacturer’s instructions and stored at -80°C until PCR amplification. The DNA extracts were used to partially amplify the 16S rDNA genes using barcoded primers 519F and 907R (Biddle et al., 2008) on the Illumina Miseq platform. Before amplification, DNA concentration and purity was monitored on 1% agarose gels. DNA was diluted to 1 ng/μL using sterile water according to the concentration. The PCR was carried out in 50-μl reaction mixtures with the following components: 4 μl (2.5 mM) of deoxynucleoside triphosphates, 2 μl (10 μM) of forward and reverse primers, 2U of Taq DNA polymerase with 0.4 μl (TaKaRa), and 1 μl of template containing approximately 50 ng of genomic community DNA as a template. Thirty-five cycles (95°C for 45 s, 56°C for 45 s, and 72°C for 60 s) were performed with a final extension at 72°C for 7 min. Triplicate reaction mixtures per sample were pooled, purified using the QIA quick PCR Purification kit (QIAGEN, Germany) and quantified using a NanoDrop ND-1000 (Thermo Scientific, United States).

Sequencing libraries were generated using TruSeq^®^ DNA PCR-Free Sample Preparation Kit (Illumina, United States) following the manufacturer’s instructions, and index codes were added. Library quality was assessed on the Qubit@ 2.0 Fluorometer (Thermo Scientific) and Agilent Bioanalyzer 2100 system. The library was sequenced on an Illumina HiSeq 2500 platform and 250 bp paired-end reads were generated.

Paired-end sequencing reads were assigned to samples based on their unique barcode and truncated by cutting off the barcode and primer sequence and then merged using FLASH (V1.2.7^1^; Magoc and Salzberg, 2011). Quality filtering on the raw tags (splicing sequences) were performed under specific filtering conditions to obtain the high-quality clean tags (Bokulich et al., 2013) using the QIIME (V1.7.0^2^; Caporaso et al., 2010) quality control process with the default parameters that sequences were quality trimmed (>25 quality score and 200 bp in length), and assigned to soil samples based on unique 5-bp barcodes. Furthermore, UCHIME algorithm (UCHIME Algorithm^3^; Edgar et al., 2011) was used to compare the tags with reference database (Gold database^4^) to detect and remove chimera sequences and then to obtain the effective tags according to Haas et al. (2011). Sequences with ≥97% similarity were assigned to the same operational taxonomic units (OTUs). Rare OTUs (less than five sequences), which could potentially have originated from sequencing errors were removed from the dataset. The most abundant sequence from each OTU was then selected as a representative sequence for that OTU. For each representative sequence, the Green Gene Database^5^ was applied to annotate taxonomic information based on RDP 3 classifier algorithm (Version 2.2^6^). At the end, OTUs abundance information was normalized using a sequence standard corresponding to the sample with the least sequences. Subsequent statistical analysis was performed basing on this output normalized data.

### Nucleotide Accession Number

The bacterial 16S rDNA genes Illumina sequencing data are deposited in the Sequence Read Archive (SRA) of National Center for Biotechnology Information (NCBI) under the BioProject number PRJNA353568.

### Statistical Analysis

Richness estimator (Chao1) and Shannon diversity index were calculated to test whether AMF inoculation and time phase influenced the bacterial OTU richness and diversity. We calculated these two indices using QIIME (Version 1.7.0) and displayed with R software (Version 2.15.3). One-way ANOVA with a general linear model (GLM) procedure (SPSS 18.0) was applied to determine the significance of different treatments (AM fungal inoculation or non-inoculation and sampling times) and their influence on the composition of the soil bacterial community. The results were expressed as mean values with standard error, and compared using Duncan’s multiple range tests. Statistical significance was determined at *P* < 0.05.

Two-way permutational multivariate analysis of variance (PERMANOVA) was carried out using the software PAST to investigate the effect of sampling time and AMF inoculation treatment on bacterial community structure (Hammer et al., 2001). Principal Component Analysis (PCA) was performed to get principal coordinates and visualizations of complex multidimensional data. A distance matrix of weighted or unweighted unifrac among samples was transformed to a new set of orthogonal axes, by which the maximum variation factor is demonstrated by the first principal coordinate, the second maximum variation factor by the second principal coordinate, and so on. PCA analysis was displayed by WGCNA package, stat packages and ggplot2 package in R software (Version 2.15.3). Goodness-of-fit statistics (*R*^2^) of measured factors fitted to the PCA ordination of the soil bacterial community were calculated using the “envfit” function in the “vegan package,” with *P*-values based on 999 permutations (Oksanen, 2007). We determined the gradient length of the soil bacterial community using Detrended Correspondence Analysis (DCA) and the results show that this bacterial community has short gradient (less than 2.7 SD) (Ramette, 2007). Thus, linear methods such as PCA are appropriate for analysis of soil bacterial community in this study.

## Results

### AM Fungal Root Colonization and Hyphae Development

Rates of root colonization by *F. mosseae* in the AMF inoculation treatment increased overtime from 24.9% at first month to 72% at fourth month and were significantly higher than control for all sampling times (Supplementary Figure S2). The control treatment had low levels of root colonization (ranged from 1.5 to 1.75%) that were not significantly different across sampling dates. Furthermore, the roots of *T. repens* remained un-nodulated for the duration of the experiment.

As a separate study we determined the soil fungal community profile in the Litter compartment using Illumina sequencing (unpublished data). These results show that the relative abundance of Glomeromycota in AMF inoculation treatment was significantly higher than control from T_90_ to T_150_ (Supplementary Figure S3). We present this data here as evidence of *F. mosseae* activity in the Litter compartment.

### Soil Bacterial Community Composition

**FIGURE 1:**
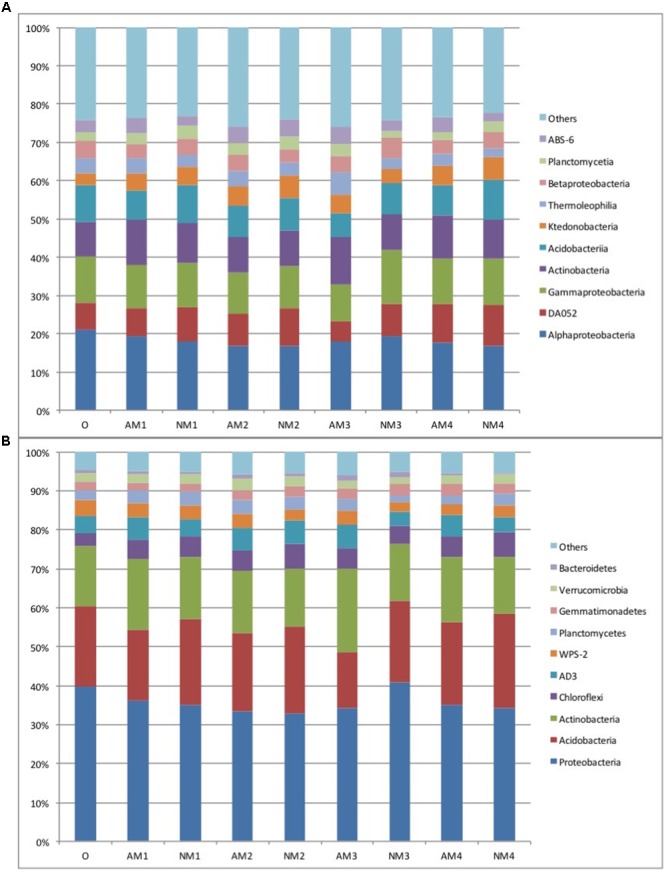
The relative abundance of the dominant bacteria class **(A)** and phyla **(B)** in soil samples derived from the different treatments and sampling times used in our study. Abbreviations: AM represents the treatment inoculated with arbuscular mycorrhizal fungi, NM represents the uninoculated treatment, while O stands for the original soil collected from the forest. Different numbers (1, 2, 3, and 4) represent the different sampling times (T_90_, T_120_, T_150_, and T_180_, respectively). Relative abundance (>1%) is based on the proportional frequencies of those DNA sequences that could be classified at the phylum or class levels. Phylogenic groups that account for less then 1% of all classified sequences are divided into the artificial group “others.”

On average, 41,385 bacterial sequences per sample survived from quality trimming and chimera removal, which were subsequently normalized to 27310 sequences per sample and assembled into 4956 OTUs with >97% similarity. We found that 729 and 217 OTUs were detected specifically in the control and AMF inoculation treatments, respectively, and 4002 OTUs were shared between treatments (for detailed OTU distribution, see Supplementary Table S1). There were eight bacterial OTUs detected in the original soil collected from the forest that were not detected in both the control and AMF inoculation treatments. Most of the obtained OTUs belonged to the phyla *Proteobacteria, Acidobacteria*, and *Actinobacteria* with a relative abundance of more than 70% per sample on average. *Chloroflexi* was the fourth most abundant phyla (5.2% relative abundance on average) followed by *Planctomycetes, Gemmatimonadete, Verrucomicrobia*, and *Bacteroidetes* (**Figure 1B**). Detailed information showing how the relative abundance of the 10 most abundant phyla changed over time and treatment has been given in Supplementary Table S2.

The relative proportion of the phyla and classes differed slightly according to sampling time and *F. mosseae* inoculation. However, the effects of *F. mosseae* inoculation on the bacterial communities were detected at a fine taxonomic resolution (genera level). The presence of AMF slightly decreased the relative abundance of *Acidobacteria* and increased that of *Actinobacteria* at each harvest, compared to the non-AM treatment (**Figure 1B**). At the class level, the 10 most abundant bacterial classes, representing over 75% of total abundance, was also slightly influenced by time and by *F. mosseae* inoculation. In each treatment, the class composition of the bacterial community showed successive changes over time (**Figure 1A**). Detailed information showing how the relative abundance of the 10 most abundant classes changed over time and treatment has been given in Supplementary Table S3. *F. mosseae* inoculation consistently increased the relative abundance of *Actinobacteria* and *Thermoleophilia* and decreased the relative abundance of *Gammaproteobacteria* and *Acidobacteria*. Additionally, the relative abundance of the most dominant class, *Alphaproteobacteria*, increased in the early stages (T_90_ and T_120_) of litter decomposition but decreased in the later stages (T_150_ and T_180_) (**Figure 1A**). While the relative abundance of the other major classes of the bacterial community fluctuated at different times and with different treatments, no consistent effects due to *F. mosseae* inoculation during litter decomposition were observed.

**FIGURE 2:**
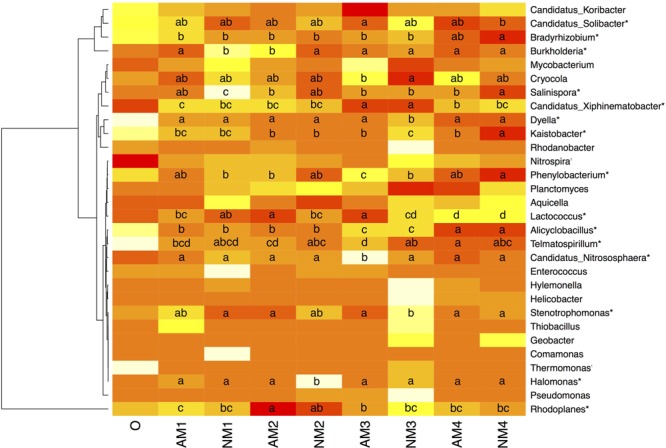
Heatmap and accompanying cluster analysis (*x*-axis) of the relative abundance of dominant bacterial genera in all the soil samples with different treatments over different sampling times. Abbreviations: AM represents the treatment inoculated with arbuscular mycorrhizal fungi, NM represents the uninoculated treatment, while O stands for the original soil collected from the forest. Different numbers (1, 2, 3, and 4) represent the different sampling times (T_90_, T_120_, T_150_, and T_180,_ respectively). Mean relative abundance is shown for each samples. For each genus, a significant difference either between different sampling times or treatments is noticed by an asterisk (*P* < 0.05). Treatments with a letter in common are not different at *P <* 0.05 according to the Duncan’s multiple range test. Differences were tested among treatment combinations (harvesting times (0 is not included) and arbuscular mycorrhizal inoculation or not) for each genus (per row). The relative abundance for each genus in each soil sample is colored in the shades of yellow (low relative abundance) to red (high relative abundance).

Analysis of the bacterial community at fine taxonomic resolution across all samples resulted in the detection of 283 genera. When conducting a significance test of the top 30 genera by relative abundance, significant changes either by time or AM fungal inoculation were found for 18 of these 30 (*P* < 0.05) (**Figure 2**). We found that *F. mosseae* inoculation significantly changed the bacterial community dynamics overtime. Specifically, in the NM treatment, the following genera demonstrated significant changes over time: *Bradyrhizobium, Burkholderia, Salinispora, Candidatus Xiphinematobacter, Dyella, Kaistobacter, Phenylobacterium, Alicyclobacillus, Halomonas*, and *Stenotrophomonas.* Furthermore, the observed changes occurred in the later stage of litter decomposition for all these genera except for *Burkholderia, Salinispora*, and *Halomonas*. Whereas changes in the relative abundance of *Salinispora and Halomonas* were only noted in the early stage of decomposition. On the other hand, in the AMF treatment, the relative abundance of *Burkholderia, Candidatus Xiphinematobacter, Phenylobacterium, Alicyclobacillus, Lactococcus, Telmatospirillum, Candidatus Nitrososphaera*, and *Rhodoplanes* were found to change significantly over the sampling time. All the noted changes occurred in the later stages of litter decomposition, while only three genera also exhibited significant changes in the early (*Burkholderia*) and both early and later stages (*Lactococcus* and *Rhodoplanes*). *Bradyrhizobium, Salinispora, Dyella, Kaistobacter, Halomonas*, and *Stenotrophomonas*, which showed significant changes overtime in NM treatment, exhibited no changes overtime in AM fungal inoculation treatment.

When comparing the difference between the two treatments, *F. mosseae* inoculation significantly changed the relative abundance of the following genera: *Burkholderia, Cryocola, Salinispora, Dyella, Kaistobacter, Phenylobacterium, Lactococcus, Telmatospirillum, Candidatus Nitrososphaera, Stenotrophomonas*, and *Halomonas* (**Figure 2**).

### The Effect of AMF on the Soil Bacterial Diversity and Richness

**FIGURE 3:**
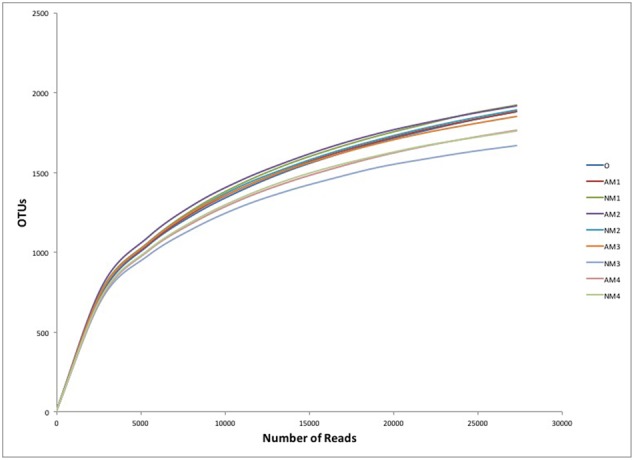
Rarefaction curve of soil bacterial OTUs clustered at 97% sequence identity across different soil samples. Abbreviations: AM represents the treatment inoculated with arbuscular mycorrhizal fungi, NM represents the uninoculated treatment, while O stands for the original soil collected from the forest. Different numbers (1, 2, 3, and 4) represent the different sampling times (T_90_, T_120_, T_150_, and T_180_, respectively).

Although we obtained high number of sequence reads per sample (27310), the rarefaction curves showed that the number of OTUs still increased with the number of sequences, without reaching a plateau (**Figure 3**). Thus, we used both Shannon diversity index and Chao1 richness estimator as proxy for bacterial diversity and richness. The presence of *F. mosseae* did not significantly change the alpha diversity and richness of the soil bacterial community, during litter decomposition. The Shannon diversity remained stable over time and across the different treatments, with an average value of 6.18 (Supplementary Figure S4A). The Chao1 richness estimator showed a similar trend with that of Shannon diversity (Supplementary Figure S4B).

We assessed the soil bacterial OTU richness at the phyla and class levels. The results indicated that *F. mosseae* did not significantly affect OTU richness at both these levels. At phylum level, the OTU-rich phyla for the NM control were phylum Proteobacteria (612 OTUs) followed by, Chloroflexi (278 OTUs), Acidobacteria (259 OTUs), Actinobacteria (222 OTUs), candidate division AD3 (38 OTUs), candidate division WPS-2 (43 OTUs). A similar pattern was also found for the OTU-rich phyla found in the AMF inoculation treatment: Proteobacteria (620 OTUs) followed by Chloroflexi (289 OTUs), Acidobacteria (259 OTUs), Actinobacteria (222 OTUs), candidate division WPS-2 (43 OTUs), and candidate division AD3 (38 OTUs). At the class level, we found that the bacterial communities from both the control and AMF inoculation treatments had the highest number of OTUs assigned to Alphaproteobacteria (control = 266 OTUs; AMF = 263 OTUs) followed by Ktedonobacteria (control = 201 OTUs; AMF = 205 OTUs), Planctomycetia (control = 194 OTUs; AMF = 196 OTUs), Gammaproteobacteria (control = 123 OTUs; AMF = 124 OTUs) and, Actinobacteria (control = 98 OTUs; AMF = 95 OTUs). Detailed changes in the richness of the 10 most abundant OTUs, for each harvest, is shown in Supplementary Table S4 (phylum level) and Supplementary Table S5 (class level).

### The Effect of AMF on the Soil Bacterial Community Structure

**FIGURE 4:**
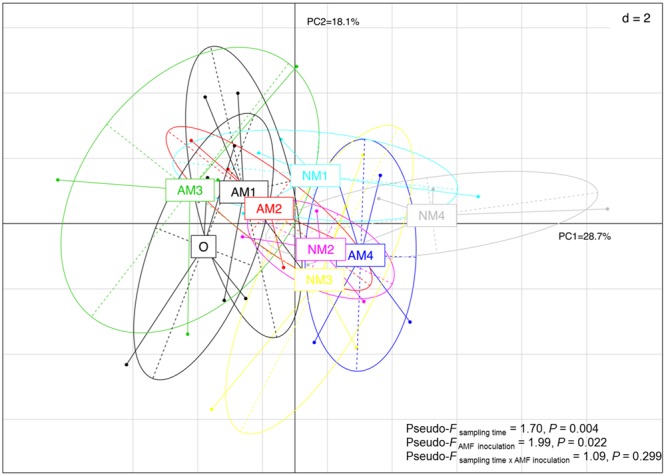
Principal component analyses (PCA) of soil bacterial community composition structure under different treatments and over different sampling times based on all the OTUs. Abbreviations: AM represents the treatment inoculated with arbuscular mycorrhizal fungi, NM represents the uninoculated treatment, while O stands for the original soil collected from the forest. Different numbers (1, 2, 3, and 4) represent the different sampling times (T_90_, T_120_, T_150_, and T_180_, respectively). The data is shown at mean value for each axis (±SE, *n* = 4).

The PCA and PERMANOVA, based on the relative abundance of all detected OTUs, showed that sampling time and AMF treatment significantly affected bacterial community composition (Pseudo-*F*_sampling time_ = 1.70, *P* < 0.004; Pseudo-*F*_AMF inoculation_ = 1.99, *P* < 0.022). The interaction between sampling time and AMF treatment was not significant (Pseudo-*F*_sampling time x AMF inoculation_= 1.09, *P* < 0.299). The PCA plot also showed that the first two canonical axes explained 28.7 and 18.1% of the total variability (**Figure 4**). The strongest effect of AMF inoculation on bacterial community structure was observed in T_150_ (**Figure 4**). Correlation analysis confirmed that AM fungal inoculation and sampling time were significantly correlated with the changes observed in the soil bacterial communities during litter decomposition. Additionally, none of the nutrients were correlated with bacterial community structure in this system (**Table 1**).

**Table 1:** Factors corresponding with soil bacterial community structure.

Factor	PC1	PC2	*R*^2^	*P*
Sampling date	0.89	–0.46	**0.20**	**0.02**
AMF inoculation	0.95	–0.32	**0.23**	**0.02**
Soil total carbon	0.09	–1.00	0.00	0.96
Soil total nitrogen	0.11	0.99	0.12	0.14
Soil total phosphate	0.74	0.67	0.03	0.61
Soil total potassium	–0.18	0.98	0.012	0.82
Soil available nitrogen	–0.58	0.82	0.05	0.45
Soil available phosphate	0.18	–0.98	0.03	0.53
Soil available potassium	0.83	–0.56	0.01	0.81
C:N	–0.10	–0.99	0.10	0.19

*P-value was based on 999 permutations. Bold font indicates significant differences (*P* < 0.05). Abbreviation: C:N (the ratio of total soil carbon to soil total nitrogen). No axis had an eigenvalue higher than 1 and the relative variance explained.*

## Discussion

Previous studies have used a variety of methods to investigate the effects of AMF on soil bacterial communities. These include PLFA analysis (Welc et al., 2010; Leifheit et al., 2015; Gui et al., 2017) and PCR-denaturing gradient gel electrophoresis (DGGE) (Marschner and Baumann, 2003; Roesti et al., 2006; Solis-Dominguez et al., 2011), as well as high-throughput methods such as 454 Pyrosequencing and Illumina sequencing (Nuccio et al., 2013; Qin et al., 2014). Our use of Illumina sequencing to test the effect of *F. mosseae* on soil bacterial communities during litter decomposition showed that although bacterial diversity and richness remained unchanged, *F. mosseae* altered the bacterial community composition and structure at different taxonomical levels during the litter decomposition process, including some key genera related to C and N cycling. We also found that the influence of *F. mosseae* on soil bacterial communities changed over time.

Although the effects of AMF on soil bacterial communities and litter decomposition have been documented in several studies (Hodge et al., 2001; Welc et al., 2010; Cheng et al., 2012; Leifheit et al., 2015), the results are not consistent between studies and the mechanisms by which AMF influences these processes remain unclear. Specifically, the relationship between AMF and bacterial richness and diversity are still largely unknown. In our study, we show that bacterial richness and diversity are not significantly affected by AMF inoculation and remain relatively stable during the litter decomposition processes. Despite a lack of difference in bacterial richness and diversity, 217 bacterial OTUs were detected in the AM treatment only, and 729 were detected in the NM treatment only. This is likely as a result of competition between the bacteria associated with AMF and other general soil bacteria, as reported in several studies (Welc et al., 2010; Nuccio et al., 2013; Mechri et al., 2014).

The bacterial community structure investigated in our study was not strongly influenced by *F. mosseae* at coarse taxonomic resolution, such as at the phylum or class levels. However, at the genus level we found that AMF had significant effects on bacterial community structure. This result is in agreement with past studies, which reported changes in the composition of soil bacterial communities as a result of AMF (e.g., Welc et al., 2010; Nuccio et al., 2013; Mechri et al., 2014). Furthermore, these observed changes were found at each sampling time, but became more pronounced over time.

The bacterial communities from the forest soils used in our study were mainly composed of Proteobacteria, Acidobacteria, Actinobacteria, and Choroflexi, regardless of the sampling time or treatment. This phylum level profile was found to be similar to the results of other studies using soil samples from different environments (Lauber et al., 2009; Li et al., 2014). Furthermore, for each sample, Proteobacteria was the most common and abundant phylum in the soil, a result that is also in line with past studies (Bastian et al., 2009; Li et al., 2014). However, our results indicated that *F. mosseae* did influence the relative abundance of these groups. For example, *F. mosseae* inoculation increased the relative abundance of Actinobacteria, and decreased the relative abundance of Acidobacteria. Actinobacteria is commonly positively correlated with AMF (Carpenter-Boggs et al., 1995; Nuccio et al., 2013), and known to inhibit the growth of other microorganisms (Duraipandiyan et al., 2010). This suppression of other organisms by Actinobacteria is the likely cause of the observed decline in the relative abundance of Acidobacteria. Furthermore, members of Proteobacteria (e.g., Alpha-, Beta-, and Gammaproteobacteria) have been identified as fast-growing bacteria that respond positively to and utilize AM fungal exudates. This could explain the observed increase in the relative abundance of Alphaproteobacteria during the initial harvests. However, a decrease in the abundance of Betaproteobacteria and Gammaproteobacteria also indicated that there was rhizosphere competition from other fast-growing bacteria such as Actinobacteria. This result agrees with that of Nuccio et al. (2013), which showed a similar decrease in the Betaproteobacterial family due to AM fungal inoculation.

At the genus level we observed that the relative abundance of certain bacterial genera were affected by *F. mosseae* inoculation. These genera shared the characteristic ability to degrade organic compounds in the soil (Xie and Yokota, 2005; Pearce et al., 2012) and included *Candidatus Solibacter, Dyella*, and *Phenylobacterium.* This result indicated that *F. mosseae* could influence litter decomposition through its effects on different bacteria in the soil; however, its effects were not uniform across genera. For example, *Candidatus Solibacter* can produce enzymes that break down organic carbon (Pearce et al., 2012). *F. mosseae* inoculation resulted in a higher relative abundance of *C. Solibacter*. Similarly, although *Dyella* and *Phenylobacterium* are both able to degrade organic compounds, *F. mosseae* inoculation positively affected the relative abundance of *Dyella* only, while the relative abundance of *Phenylobacterium* was negatively affected during the later stages of decomposition. Furthermore, our results indicate that bacteria from the genus *Burkholderia*, a rhizosphere-colonizing bacteria with saprophytic abilities (Roberts et al., 1997), were suppressed at T_90_. We also found that *Geobacter*, which is able to oxidize organic compounds (Childers et al., 2002) was abundant throughout the decomposition process, although its relative abundance was not affected by *F. mosseae* inoculation.

Our findings agree with those of Ravnskov et al. (2002), who reported that *Rhizophagus irregularis* suppressed the growth and development of *Burkholderia cepacia* populations. However, Mansfeld-Giese et al. (2002) showed that the external mycelia of *F. intraradices* increased the population of *Pseudomonas chlororaphis* in the soil, whereas in our experiment *Pseudomonas* abundance did not change significantly with *F. mosseae* inoculation. Our work supports past findings showing that *Pseudomonas* is frequently detected in litter decomposition processes (Noll et al., 2010).

## Conclusion

Our study confirms previous reports on the influence of AMF on soil bacterial communities and provides detailed insight into the changes that occur in bacterial communities due to the presence of AMF. In addition, we were able to show how the influence of AMF on bacterial communities changes over time. AMF significantly altered the bacterial community dynamics and structures at fine taxonomic resolution, with no noticeable changes occurring at the phylum and class levels. Our work demonstrates that analysis of bacterial communities using high-resolution culture independent methods can significantly improve our understanding of bacterial richness, diversity and community dynamics in complex soil environments.

## Author Contributions

HG carried out the experiment, performed the data analysis, and took lead on writing the manuscript. PM developed the original ideas, oversaw the research work, and contributed toward writing the manuscript. WP contributed toward data analysis and manuscript revision. JX and KH contributed toward writing the manuscript.

## Conflict of Interest Statement

The authors declare that the research was conducted in the absence of any commercial or financial relationships that could be construed as a potential conflict of interest.

## Footnotes

1 http://ccb.jhu.edu/software/FLASH/

2 http://qiime.org/index.html

3 http://www.drive5.com/usearch/manual/uchime_algo.html

4 http://drive5.com/uchime/uchime_download.html

5 http://greengenes.lbl.gov/cgi-bin/nph-index.cgi

6 http://sourceforge.net/projects/rdp-classifier/
