# Supplementary material for: The Arbuscular Mycorrhizal Fungus Funneliformis mosseae Alters Bacterial Communities in Subtropical Forest Soils during Litter Decomposition
Source: Front Microbiol. 2017 Jun 20;8:1120. doi: 10.3389/fmicb.2017.01120 (PMC5476864; doi:10.3389/fmicb.2017.01120)
Supplement: Supplementary file 1 [file Data_Sheet_1.DOCX]

**Supplemental Material**

**The arbuscular mycorrhizal fungus *Funneliformis mosseae* alters soil bacterial communities in subtropical forest soils during litter decomposition**

**Heng Gui^1,2,3,4^, Witoon Purahong^5^, Kevin Hyde^3,4^, Jianchu Xu^1,2^, Peter Mortimer^1,2*^**

^1^Key laboratory for Plant Diversity and Biogeography of East Asia, Kunming Institute of Botany, Chinese Academy of Science, Kunming 650201, China

^2^World Agroforestry Centre, East and Central Asia, Kunming 650201, China

^3^Centre of Excellence in Fungal Research, Mae Fah Luang University, Chiang Rai 57100, Thailand

^4^School of Science, Mae Fah Luang University, Chiang Rai 57100, Thailand

^5^Department of Soil Ecology, UFZ-Helmholtz Centre for Environmental Research, Halle (Saale) D-06120, Germany

**Figure S1.** Schematic structure of the split-root microcosm

**Figure S2.** AM fungal colonization rate (%) at different harvest times (AM+ represents for AM fungal inoculation and AM- represents for non-inoculation, n=4, Mean±SE, *indicates significant different between AM+ and AM- treatments, *P* < 0.05)

Figure S3. The relative abundance of phylum Glomeromycota over different harvest times (T_90_, T_120_, T_150_, and T_180_) and different treatment (AM+ and AM-). The data was shown as Mean±SE (n=4). AM+ represents for AM fungal inoculation and AM- represents for non-inoculation, *indicates significant different between AM+ and AM- treatments (*P* < 0.05).


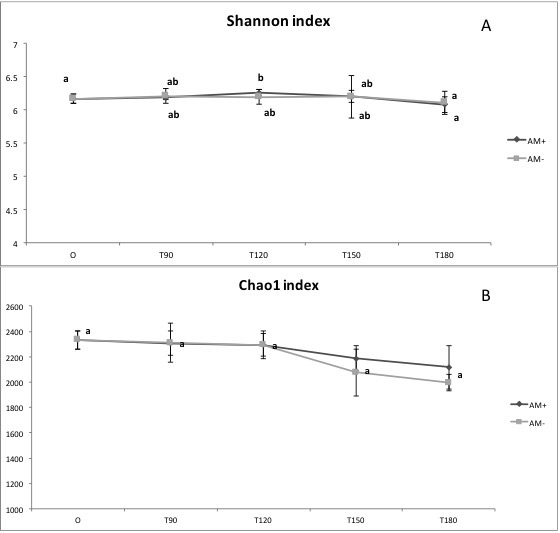


**Figure S4.** The alpha-diversity of soil bacterial community as indicated by Shannon (A) and Chao1 (B) indices, under different treatments (AM+ and AM-) and over different sampling times (T90, T120, T150, and T180 respectively), based on all the OTUs. Data are presented as means ± SE (n=4). Abbreviations: AM+ represents the treatment inoculated with arbuscular mycorrhizal fungi, AM- represents the uninoculated treatment, while 0 stands for the original soil collected from the forest. Different letters indicate significant differences (*P*<0.05).

**Table S1.** The detailed taxonomical information of OTUs shared by AM and NM, specific AM OTUs and specific NM OTUs. AM: AM fungal inoculation treatment, NM: non-inoculation control, N/A: no available taxonomical information at this level. (uploaded as an individual excel file)

Table S2. The changes in relative abundance of the 10 most abundant phyla for the different treatments (AM and NM) and sampling times.

| Phylum | O | AM1 | NM1 | AM2 | NM2 | AM3 | NM3 | AM4 | NM4 |
| --- | --- | --- | --- | --- | --- | --- | --- | --- | --- |
| Proteobacteria | 0.397±0.062 | 0.36±0.029 | 0.35±0.034 | 0.334±0.011 | 0.329±0.019 | 0.341±0.067 | 0.409±0.055 | 0.35±0.039 | 0.343±0.049 |
| Acidobacteria | 0.207±0.024 | 0.183±0.029 | 0.22±0.058 | 0.201±0.041 | 0.223±0.034 | 0.144±0.031 | 0.21±0.037 | 0.214±0.029 | 0.242±0.072 |
| Actinobacteria | 0.152±0.019 | 0.182±0.041 | 0.159±0.017 | 0.158±0.036 | 0.148±0.022 | 0.214±0.033 | 0.145±0.035 | 0.166±0.033 | 0.146±0.018 |
| Chloroflexi | 0.035±0.01 | 0.05±0.014 | 0.052±0.005 | 0.054±0.006 | 0.063±0.018 | 0.053±0.034 | 0.046±0.021 | 0.054±0.019 | 0.061±0.012 |
| AD3 | 0.042±0.002 | 0.056±0.006 | 0.044±0.004 | 0.057±0.004 | 0.059±0.008 | 0.061±0.011 | 0.037±0.006 | 0.055±0.007 | 0.039±0.004 |
| WPS-2 | 0.042±0.016 | 0.038±0.005 | 0.038±0.004 | 0.038±0.012 | 0.029±0.005 | 0.034±0.011 | 0.024±0.004 | 0.027±0.002 | 0.031±0.006 |
| Planctomycetes | 0.025±0.015 | 0.03±0.005 | 0.035±0.005 | 0.035±0.004 | 0.033±0.011 | 0.033±0.019 | 0.017±0.01 | 0.022±0.008 | 0.03±0.007 |
| Gemmatimonadetes | 0.023±0.004 | 0.02±0.003 | 0.019±0.001 | 0.023±0.009 | 0.025±0.002 | 0.025±0.01 | 0.031±0.009 | 0.03±0.006 | 0.026±0.005 |
| Verrucomicrobia | 0.021±0.01 | 0.023±0.004 | 0.025±0.003 | 0.03±0.004 | 0.026±0.001 | 0.021±0.009 | 0.017±0.004 | 0.022±0.004 | 0.023±0.004 |
| Bacteroidetes | 0.008±0.002 | 0.007±0.001 | 0.006±0.002 | 0.011±0.003 | 0.007±0.002 | 0.013±0.006 | 0.013±0.011 | 0.005±0.001 | 0.004±0.002 |
| Others | 0.047±0.008 | 0.05±0.001 | 0.053±0.008 | 0.059±0.004 | 0.056±0.006 | 0.061±0.013 | 0.051±0.012 | 0.056±0.008 | 0.055±0.005 |

Abbreviations: AM represents the treatment inoculated with arbuscular mycorrhizal fungi, NM represents the uninoculated treatment, while O stands for the original soil collected from the forest. Different numbers (1, 2, 3, and 4) represent the different sampling times (T_90_, T_120_, T_150_, and T_180_ respectively). The data were shown at the mean value (±SE, n=4).

Table S3. The changes in relative abundance of the 10 most abundant classes for the different treatments (AM and NM) and sampling times.

| Class | O | AM1 | NM1 | AM2 | NM2 | AM3 | NM3 | AM4 | NM4 |
| --- | --- | --- | --- | --- | --- | --- | --- | --- | --- |
| Alphaproteobacteria | 0.211±0.036 | 0.193±0.02 | 0.178±0.017 | 0.168±0.008 | 0.167±0.014 | 0.179±0.03 | 0.193±0.042 | 0.176±0.024 | 0.167±0.024 |
| DA052 | 0.07±0.013 | 0.072±0.013 | 0.091±0.036 | 0.085±0.026 | 0.099±0.017 | 0.054±0.015 | 0.085±0.022 | 0.101±0.016 | 0.109±0.047 |
| Gammaproteobacteria | 0.121±0.02 | 0.114±0.011 | 0.116±0.006 | 0.106±0.004 | 0.11±0.015 | 0.095±0.027 | 0.139±0.023 | 0.118±0.015 | 0.12±0.008 |
| Actinobacteria | 0.09±0.02 | 0.119±0.033 | 0.105±0.011 | 0.094±0.021 | 0.093±0.015 | 0.124±0.014 | 0.093±0.034 | 0.113±0.027 | 0.101±0.011 |
| Acidobacteriia | 0.096±0.013 | 0.076±0.009 | 0.097±0.021 | 0.081±0.012 | 0.085±0.013 | 0.061±0.02 | 0.082±0.016 | 0.08±0.006 | 0.106±0.025 |
| Ktedonobacteria | 0.031±0.009 | 0.046±0.013 | 0.048±0.005 | 0.05±0.006 | 0.059±0.017 | 0.049±0.034 | 0.037±0.015 | 0.05±0.018 | 0.058±0.011 |
| Thermoleophilia | 0.038±0.003 | 0.04±0.006 | 0.033±0.005 | 0.041±0.01 | 0.033±0.007 | 0.058±0.018 | 0.03±0.004 | 0.031±0.003 | 0.024±0.004 |
| Betaproteobacteria | 0.046±0.01 | 0.035±0.003 | 0.041±0.013 | 0.04±0.009 | 0.034±0.003 | 0.042±0.015 | 0.054±0.012 | 0.037±0.003 | 0.041±0.018 |
| Planctomycetia | 0.024±0.015 | 0.03±0.005 | 0.033±0.005 | 0.034±0.004 | 0.032±0.011 | 0.031±0.018 | 0.015±0.007 | 0.021±0.008 | 0.029±0.007 |
| ABS-6 | 0.031±0.003 | 0.04±0.008 | 0.027±0.004 | 0.042±0.003 | 0.046±0.008 | 0.045±0.009 | 0.029±0.005 | 0.039±0.008 | 0.021±0.004 |
| Others | 0.242±0.021 | 0.236±0.008 | 0.231±0.02 | 0.259±0.012 | 0.24±0.007 | 0.259±0.006 | 0.243±0.035 | 0.234±0.016 | 0.224±0.022 |

Abbreviations: AM represents the treatment inoculated with arbuscular mycorrhizal fungi, NM represents the uninoculated treatment, while O stands for the original soil collected from the forest. Different numbers (1, 2, 3, and 4) represent the different sampling times (T_90_, T_120_, T_150_, and T_180_ respectively). The data were shown at the mean value (±SE, n=4).

Table S4. The 10 bacterial groups with the greatest OTU richness at the phylum level, for different treatments (AM and NM) and sampling times.

| OTU richness | O | AM1 | NM1 | AM2 | NM2 | AM3 | NM3 | AM4 | NM4 |
| --- | --- | --- | --- | --- | --- | --- | --- | --- | --- |
| Proteobacteria | 698 | 618 | 625 | 637 | 630 | 589 | 609 | 634 | 584 |
| Acidobacteria | 283 | 260 | 268 | 265 | 263 | 250 | 255 | 263 | 253 |
| Actinobacteria | 228 | 219 | 233 | 228 | 218 | 220 | 219 | 222 | 219 |
| Chloroflexi | 261 | 283 | 309 | 311 | 294 | 290 | 228 | 267 | 279 |
| AD3 | 37 | 37 | 39 | 40 | 39 | 39 | 36 | 36 | 38 |
| WPS-2 | 44 | 45 | 47 | 45 | 41 | 42 | 41 | 41 | 43 |
| Planctomycetes | 236 | 283 | 281 | 295 | 281 | 273 | 170 | 230 | 255 |
| Gemmatimonadetes | 45 | 39 | 41 | 39 | 42 | 42 | 40 | 49 | 38 |
| Verrucomicrobia | 53 | 55 | 53 | 58 | 57 | 53 | 50 | 55 | 52 |
| Bacteroidetes | 43 | 45 | 42 | 40 | 41 | 39 | 42 | 42 | 40 |

Abbreviations: AM represents the treatment inoculated with arbuscular mycorrhizal fungi, NM represents the uninoculated treatment, while O stands for the original soil collected from the forest. Different numbers (1, 2, 3, and 4) represent the different sampling times (T_90_, T_120_, T_150_, and T_180_ respectively). The data were shown at the mean value.

Table S5. The 10 bacterial groups with the greatest OUT richness at the class level, for different treatments (AM and NM) and sampling times.

| OTU richness | O | AM1 | NM1 | AM2 | NM2 | AM3 | NM3 | AM4 | NM4 |
| --- | --- | --- | --- | --- | --- | --- | --- | --- | --- |
| Alphaproteobacteria | 284 | 270 | 268 | 272 | 268 | 258 | 280 | 253 | 247 |
| DA052 | 54 | 54 | 56 | 55 | 54 | 51 | 54 | 57 | 54 |
| Gammaproteobacteria | 135 | 124 | 123 | 126 | 120 | 124 | 132 | 123 | 118 |
| Actinobacteria | 95 | 101 | 103 | 93 | 96 | 97 | 100 | 92 | 95 |
| Acidobacteriia | 85 | 82 | 84 | 82 | 83 | 79 | 81 | 80 | 82 |
| Ktedonobacteria | 188 | 209 | 228 | 216 | 217 | 206 | 162 | 191 | 201 |
| Thermoleophilia | 46 | 44 | 46 | 45 | 45 | 48 | 48 | 43 | 43 |
| Betaproteobacteria | 45 | 39 | 41 | 41 | 40 | 40 | 51 | 41 | 37 |
| Planctomycetia | 188 | 208 | 222 | 214 | 219 | 191 | 142 | 172 | 196 |
| ABS-6 | 11 | 13 | 11 | 14 | 13 | 13 | 11 | 12 | 10 |

Abbreviations: AM represents the treatment inoculated with arbuscular mycorrhizal fungi, NM represents the uninoculated treatment, while O stands for the original soil collected from the forest. Different numbers (1, 2, 3, and 4) represent the different sampling times (T_90_, T_120_, T_150_, and T_180_ respectively). The data were shown at the mean value.
